# Stepped-wedge randomised trial of laparoscopic ventral mesh rectopexy in adults with chronic constipation: study protocol for a randomized controlled trial

**DOI:** 10.1186/s13063-018-2456-3

**Published:** 2018-02-05

**Authors:** Ugo Grossi, Natasha Stevens, Eleanor McAlees, Jon Lacy-Colson, Steven Brown, Anthony Dixon, Gian Luca Di Tanna, S. Mark Scott, Christine Norton, Nadine Marlin, James Mason, Charles H. Knowles, On behalf of the NIHR CapaCiTY working group, Mark Chapman, Andrew Williams, Mark Mercer-Jones, Karen Telford, Andrew Clarke, Sophie Pilkington, Yan Yiannakou, Neil Smart, Douglas Tincello, Andrew Miller, Kenneth Campbell, Neil Cruickshank, Christopher Emmett, David Pares, Emma Horrocks, Paul Vollebregt, Ian Lindsey

**Affiliations:** 1 National Bowel Research Cente (NBRC) – Digestive Disease, Barts and The London School of Medicine and Dentistry, Queen Mary, University of London, 4 Newark Street, London, E1 2AT UK; 2 Pragmatic Clinical Trials Unit, Blizard Institute, Queen Mary, University of London, London, UK; 3 Royal Shrewsbury Hospital, Shrewsbury, UK; 4 Northern General Hospital, Sheffield, UK; 5 University of Bristol, Bristol, UK; 6 King’s College London, London, UK; 7 University of Warwick, Coventry, UK

**Keywords:** CapaCiTY, CapaCiTY study 3, Chronic constipation, Laparoscopic ventral mesh rectopexy (LVMR), Rectopexy, Internal rectal prolapse, Surgery, Stepped wedge

## Abstract

**Background:**

Laparoscopic ventral mesh rectopexy (LVMR) is an established treatment for external full-thickness rectal prolapse. However, its clinical efficacy in patients with internal prolapse is uncertain due to the lack of high-quality evidence.

**Methods:**

An individual level, stepped-wedge randomised trial has been designed to allow observer-blinded data comparisons between patients awaiting LVMR with those who have undergone surgery. Adults with symptomatic internal rectal prolapse, unresponsive to prior conservative management, will be eligible to participate. They will be randomised to three arms with different delays before surgery (0, 12 and 24 weeks). Efficacy outcome data will be collected at equally stepped time points (12, 24, 36 and 48 weeks). The primary objective is to determine clinical efficacy of LVMR compared to controls with reduction in the Patient Assessment of Constipation Quality of Life (PAC-QOL) at 24 weeks serving as the primary outcome. Secondary objectives are to determine: (1) the clinical effectiveness of LVMR to 48 weeks to a maximum of 72 weeks; (2) pre-operative determinants of outcome; (3) relevant health economics for LVMR; (4) qualitative evaluation of patient and health professional experience of LVMR and (5) 30-day morbidity and mortality rates.

**Discussion:**

An individual-level, stepped-wedge, randomised trial serves the purpose of providing an untreated comparison for the active treatment group, while at the same time allowing the waiting-listed participants an opportunity to obtain the intervention at a later date. In keeping with the basic ethical tenets of this design, the average waiting time for LVMR (12 weeks) will be shorter than that for routine services (24 weeks).

**Trial registration:**

ISRCTN registry, ISRCTN11747152. Registered on 30 September 2015. The trial was prospectively registered (first patient enrolled on 21 March 2016).

**Electronic supplementary material:**

The online version of this article (10.1186/s13063-018-2456-3) contains supplementary material, which is available to authorized users.

## Background

### Burden of disease

Constipation is common in adults and children and up to 20% of the population report this symptom depending on definitions used (2–28% adults; 0.7–30% children) [1–3], with a higher prevalence in women [1, 4, 5] and the elderly [6, 7]. Chronic constipation (CC), usually defined as more than 6 months of symptoms, is less common [8] but results in 0.5 million UK General Practitioner (GP) consultations per annum. A proportion of the population suffers symptoms that are both chronic and more disabling (probably about 1–2% of the population) [9]. Such patients, who are very frequently female [10], are usually referred to secondary care with many progressing to tertiary specialist investigation. Patient dissatisfaction is high in this group; nearly 80% feel that laxative therapy is unsatisfactory [11] and the effect of symptoms on measured quality of life (QOL) is significant [12]. CC consumes significant healthcare resources. In the USA in 2012, a primary complaint of constipation was responsible for 3.2 million physician visits [13] resulting in (direct and indirect) costs of US$1.7 billion. In the UK, it is estimated 10% of district nursing time is spent on constipation [14] and the annual spend on laxatives exceeds £80 million, with a cost of £17.4 million for prescriptions in 2012 (Health and Social Care Information Centre, 2013) [15].

### Pathophysiological basis of chronic constipation

The act of defaecation is dependent on the coordinated functions of the colon, rectum and anus. Considering the complexity of neuromuscular (sensory and motor) functions required to achieve planned, conscious, and effective defaecation [16], it is no surprise that disturbances to perceived “normal” function occur commonly at all stages of life. Clinically, such problems commonly lead to symptoms of obstructed defaecation (e.g. straining; incomplete, unsuccessful or painful evacuation; bowel infrequency and abdominal pain and bloating). After exclusion of a multitude of secondary causes (obstructing colonic lesions, neurological, metabolic and endocrine disorders), the pathophysiology of CC can broadly be divided into problems of colonic contractile activity and thus stool transit and problems of the pelvic floor. Thus, with specialist radio-physiological investigations (hereafter referred to as INVEST in this protocol), patients may be divided into those who have slow colonic transit, evacuation disorder, both or neither (no abnormality found with current tests). Evacuation disorders can be then subdivided into those in which a structurally significant pelvic-floor abnormality is evident, e.g. rectocele or internal prolapse (intussusception), and those in which there is a dynamic failure of evacuation without structural abnormality, most commonly termed functional defaecation disorder (FDD).

### Chronic constipation management overview

Management of CC is a major problem due to its high prevalence and lack of widespread specialist expertise. In general, a step-wise approach is undertaken, with first-line conservative treatment such as lifestyle advice and laxatives (primary care) followed by nurse-led bowel re-training programmes, sometimes including focused biofeedback and psychosocial support (secondary/tertiary care). Although these treatments may improve symptoms in more than half of patients, they are very poorly standardised in the UK and are not universally successful. Patients with intractable symptoms and impaired QOL may subsequently be offered a range of costly, irreversible surgical interventions with unpredictable results [17, 18], sometimes resulting in major adverse events or a permanent stoma.

### Overall rationale for the CapaCiTY programme

The current trial forms part of a National Institute for Health Research (NIHR)-funded programme (PGfAR, RP-PG-0612-20001). This programme aims to develop the evidence base for the management of CC in adults, which is currently lacking. This is in contrast to the management of CC in children for which National Institute for Health and Care Excellence (NICE) guidance has been recently published (https://www.nice.org.uk/guidance/cg99) [19, 20], and for adults with faecal incontinence (http://pathways.nice.org.uk/pathways/faecal-incontinence). Thus there are considerable variations in practice, particularly in specialist services. With a number of new drugs gaining or seeking National Health Service (NHS) approval [21–24] and technologies at a horizon scanning stage [17, 25–27], it is timely that the currently limited evidence base is developed for resource-constrained NHS providers to have confidence that new and sometimes expensive investigations and therapies are appropriate and cost-effective. A cost-conscious pathway of care may help reduce healthcare expenditures by appropriately sequencing the care provided, while targeting more expensive therapies at those most likely to benefit. Such data will inform the development and commissioning of integrated care pathways. An overview of the CapaCiTY programme is provided as a scheme (see Additional file 1) and includes a series of interlinked studies that answer the important questions for patient care. A rolling programme of national recruitment will provide a large cohort of well-defined patients for three subsequent studies over 5 years. The focus will be on generating real-life evidence from pragmatic studies that will provide valid clinical outcome measures, and address patient acceptability and cost. Armed with such data it will be possible to develop an NHS management algorithm for CC, which will meet patient, clinician and policy aims.

### Specific clinical background to the trial of laparoscopic ventral mesh rectopexy (LVMR)

In most UK practices, patients are first referred to specialist nurses for a variety of nurse-led behavioural interventions to improve defaecatory function. A range of cohort studies [28], randomized controlled trials (RCTs) [29–34], reviews [35], guidelines [36], a meta-analysis [37] and a Cochrane review [38] attest to the general success of this approach. Specific methodological issues are being addressed by CapaCiTY study 1. Patients failing behavioural interventions may progress to anal irrigation (CapaCiTY study 2). However despite these approaches, some patients will have persistent intractable symptoms.

When non-surgical therapies fail, a decision must be made whether to offer surgical intervention. Decision-making is greatly influenced by local expertise, commissioning and personal enthusiasm for particular interventions [17, 18, 39], balanced against poor results in some patients [17]. Currently, there is thus large and difficult-to-justify variation in surgical practice according to need and type of procedure. The need to reduce variations in practice, based on available evidence, has been a perpetual theme of recent national speciality group discussions [40] with various initiatives proposed. A Multidisciplinary Decision Team (MDT), incorporating expertise from nurses, gastroenterologists, urogynaecologists, colorectal surgeons and psychologists to promote appreciation of the whole pelvic floor (bladder, vagina, uterus and bowel), could reduce the potential for inadequately-informed and potentially harmful interventions in poor surgical candidates [17], but the utility of this approach has not been formally tested. Further, there are few data on outcomes in well-characterised patient cohorts or rational criteria for patient selection.

In practice, there are few pelvic-floor procedures that are commonly employed in patients with CC, these being forms of rectopexy and rectocele repair in conjunction with urogynaecological approaches to other organ prolapse [41]. Other procedures are only occasionally performed in highly selected patients (e.g. colectomy/ileostomy [18]), or should only be performed on a research protocol basis (e.g. stapled transanal resection [36]) or are subject to specialist commissioning approval (e.g. sacral nerve stimulation [25, 27]).

LVMR is established as a treatment for external full-thickness rectal prolapse [42–44] but is now being widely performed internationally (including in many centres in the UK) on large numbers of patients with defaecatory problems concomitant with evidence of pelvic-floor weakness - mainly rectocele and intussusception [45–52]. The evidence needs for LVMR relate to the following observations:

1. Patient selection:
  Rectocele and intussusception are present in at least 40% of asymptomatic women, detection depends significantly on method of assessment [53, 54] and they frequently co-exist [55].
  The evident structural abnormality often belies a complex multifactorial problem with several contributing types of aetiology that cannot be addressed by surgery alone [56].
  Structural correction (by a variety of approaches) often poorly correlates with functional outcomes [57–59].
2. Lack of trial evidence of efficacy:
  Evidence is based solely on short-term observational data obtained in the most part from individual expert case series [51, 60–62] and to some extent by evolving patient registries (populated by the same experts).
  Outcomes have generally been based on poorly validated measures (e.g. patient global rating scales [63]) and some bespoke summative scores (e.g. obstructive defecation score [ODS] [45, 64]), which were originally developed to show the benefit of surgery [65, 66].
  There is concern that objectively determined long-term outcomes of LVMR using validated measures will not match those from enthusiastically driven case series (as observed for numerous other surgical procedures with the intent of addressing CC) [17].
3. Risk:
  While early data show that LVMR is relatively safe from immediate complications, it is acknowledged that the placement of a mesh in the pelvis is a high risk strategy due to problems of migration, infection and erosion [67]. The use of mesh placed trans-vaginally has now led to class actions in all states of the USA amounting to billions of dollars of law-suits (http://www.drugwatch.com/transvaginal-mesh/lawsuit.php). Several countries (including Scotland) (http://www.bbc.co.uk/news/uk-scotland-scotland-politics-27884794) have suspended its use on this basis. While placement of mesh trans-abdominally is recognised to be safer (no exposure to vaginal bacterial flora), and biological mesh may reduce this complication (compared to synthetic mesh) [68–70], there are still reported morbidity rates in the 1–2% range [70, 71].
  As with all other pelvic floor operations, some patients may be made functionally worse by surgery due to worsening of evacuation problems, new problems of incontinence caused by altered pelvic floor dynamics [72] and chronic pelvic pain or dyspareunia [70]. Such problems are then very difficult to correct by any method.

Such is the debate on LVMR that almost all international coloproctology meetings have whole sessions dedicated to its discussion (especially the issue of mesh complications); a recent consensus report has also been published [73]. It is clear that while these complications may be limited by good technique and perhaps choice of mesh, they will not be eradicated. Thus, it can be argued that the future of LVMR depends not on the very small observed differences in long-term mesh complications (e.g. 1.0–2.0% of patients) but on a fundamental evaluation of whether the procedure is actually clinically beneficial, i.e. whether these complication rates would be deemed acceptable (provided patients are consented to the risk) if the patient benefit was sufficiently large. The aim of the proposed trial is to address this knowledge gap.

### Specific study rationale

The overall rationale is to address the main objectives (see below) within a controlled trial. We have used a stepped-wedge, randomised trial design which permits observer-masked data comparisons between patients awaiting intervention with those who have undergone surgery. Contrary to most stepped-wedge trials individual patients are randomised rather than clustered. In brief (more detail below), eligible participants based on clinical evaluation and radio-physiological investigations (INVEST) will be randomised to three arms with different lengths of delay before surgery (see Additional file 2). In all arms there will be a period of 4 weeks post-eligibility to arrange the logistics of surgery (time (T)–4 weeks to T0) and ensure that patients have returned to their normal life routine after various assessments. LVMR will be performed at T0 in group I; T12 (12 weeks) in group II and T24 (24 weeks) in group III. Unavoidably, participants will be aware when surgery is undertaken: this, however, fortuitously meets the assumptions of the stepped-wedge design, i.e. no effect of treatment is expected until surgery has been performed. Efficacy outcome data will be collected at equally stepped time points (T0, 12, 24, 36 and 48 weeks).

This is, in effect, a modification of a standard, parallel-arm, wait-list control design, but with several advantages. First, a stepped-wedge design is more efficient and thus improves recruitment feasibility (the bane of nearly all surgical trials). Despite the multicentre approach of this study, the problems of recruitment cannot be underestimated. Simulation demonstrates that a parallel-arm design requires a much larger sample size than that proposed for the current study at the same power. Secondly, the trial design means that there is only a one in three chance (rather than one in two for a parallel arm) of waiting 6 months for surgery, which is more acceptable to patients.

### Risks and benefits of participation

The risks of trial participation are considered very low over and above standard surgical risks. The intervention proposed is already offered to patients in specialist centres throughout the UK and internationally. The only difference conferred by participation is that the intervention will be randomly allocated by time and very carefully assessed. CC is a chronic condition (especially by the time conservative treatments have failed) and thus allocation to waiting times of up to 24 weeks poses no clinical risk. Radio-physiological tests (INVEST) are required to select patients with appropriate structural pelvic-floor problems for surgery. These would be performed in routine clinical practice in all patients undergoing LVMR and will also be mandated for the trial using specified techniques and equipment. While this may lead to slight variance from normal practice, the fundamental tests and their safety remain unchanged. Such tests have been performed daily in most specialist centres for up to 30 years without any recorded complication (Barts Health experience is over 10,000 patients). A small ionising radiation dose is required for two tests (covered below). A number of questionnaires contain personal questions about bowel problems and the effect of these on QOL and psycho-behavioural functioning; however, all have been used in studies of similar patients previously. The design of the study requires data collection at time points additional to those required for the analysis of the primary and secondary endpoints. However, this streamlines the logistics and management of the study participants through the course of the study whilst ensuring blinding is maintained and eliminating observer bias. This small additional burden on participants has been carefully balanced against the obvious benefits of the design and efficiencies of sample size gained, reducing the overall number of participants required to undergo surgery.

The benefits of participation are that patients will receive a very high standard of surgery (the most experienced UK surgeons will be participating). Further, by design, the fidelity of surgical technique will be standardised and tightly scrutinised (including by preceptorship and mentorship if required); they will also receive a high standard of monitored care as a consequence of the detailed protocol.

## Design/methods

This is a stepped-wedge, randomised trial of LVMR in adults with chronic constipation (*n* = 114), which follows the “Standard Protocol Items: Recommendations for Interventional Trials” (SPIRIT) guidelines (see Additional file 3). Participants will be randomized to three equal arms (*n* = 38) with different lengths of delay before surgery. LVMR will be performed at T0 in group I; T12 (12 weeks) in group II and at T24 (24 weeks) in group III.

### Trial objectives and endpoints

#### Primary objective

The primary objective is to determine the clinical efficacy of LVMR compared to controls at short-term follow up (24 weeks).

#### Secondary objectives

The secondary objectives are to:

1. Determine the clinical effectiveness of LVMR in the medium term (to 48 weeks to a maximum of 72 weeks)
2. Determine pre-operative determinants of the outcome
3. Determine relevant health economics for LVMR
4. Qualitatively evaluate patient and health professional experience of LVMR
5. Assess 30-day morbidity and mortality rates

### Trial outcomes

#### Primary outcome

The primary clinical efficacy endpoint is based on the Patient Assessment of Constipation-Quality of Life (PAC-QOL) questionnaire total score (analysed as a continuous variable) in patients at 24 weeks post-surgery compared to pre-surgery controls. The secondary clinical efficacy endpoints are based on the Patient Assessment of Constipation Symptoms (PAC-SYM) questionnaire total score (analysed as a continuous variable) in patients at 24 weeks post-surgery compared to pre-surgery controls.

#### Secondary outcomes

All outcomes within the standardised outcome framework will be analysed to compare baseline values with values at 24 and 48 weeks post-surgery follow up. When further follow-up data are obtained (time permitting) these will also be reported at the later time points of 60 and 72 weeks. The outcomes are:

- Response to treatment defined as a 1-point (or greater) reduction in the PAC-QOL score [74, 75].
- PAC-QOL: individual domains and total score (as continuous variables).
- PAC-SYM score: individual domains and total score (as continuous variables).
- A 2-week patient diary (for 2 weeks prior to each assessment) to record bowel frequency and whether each evacuation was “spontaneous (no use of laxatives) and/or complete”; a journal will also capture concurrent medication, health contacts, time away from normal activities (including work) since the patient’s last visit.
- Generic QOL assessment: EuroQol Health Outcome measure (EQ-5D)-5L descriptive system and EQ-visual analogue scale (VAS) [76]. Note: the EQ-VAS has SD of approximately 30 points: a 10% difference in VAS deemed clinically significant can be detected with the large sample sizes proposed.
- Patient Health Questionnaire-9 (PHQ-9) [58, 59].
- Generalized anxiety disorder questionnaire (GAD7) [60].
- Global patient satisfaction/improvement score (VAS) and whether they would recommend LVMR to other patients.
- Potentially modifiable cognitive and behavioural psychological variables shown to predict onset and perpetuation of other functional bowel symptoms: negative perfectionism [71], avoidant and “‘all or nothing” behaviour subscales of the behavioural response to illness questionnaire (CC-BRQ) [77], and brief illness perception questionnaire (BIPQ) (CC) [78].
- St Marks Incontinence score (for concurrent symptoms) [79].
- Baseline brief sexual function questionnaire (Pelvic Organ Prolapse/Urinary Incontinence Sexual Questionnaire (PISQ-12) in women [80] and Male Sexual Health Questionnaire (MSHQ)-EjD Short Form in men [81]).

#### Specific adverse events and surgery-specific data

Data will be collected on:

- Perioperative findings, e.g. scarring, adhesions, tissue laxity, pelvic depth, ovarian or uterine pathology
- Procedural data: duration of surgery, blood loss, approach (laparoscopic vs. conversion to open), type of mesh and sutures (make, diameter, number)
- Length of post-operative stay
- Re-admission at 30 days
- Complications: 30-day morbidity and mortality
- Specific outcomes, e.g. dyspareunia and sexual function, pelvic pain, urinary dysfunction, new onset of faecal incontinence or early mesh complications (displacement, erosion, infection)
- Early clinical recurrence of structural defect e.g. prolapse or rectocele based on rectal examination with/without adjunctive investigations (as clinically indicated)

### Study setting

The study setting is in specialist centres across the UK with surgical expertise in LVMR; approximately 10 NHS Trusts will recruit patients into the study. Eligibility of the surgeons is based on having previously performed a minimum of 50 LVMR and undergoing independent assessment of adherence to the defined LVMR procedural sequences.

### Recruitment

Patients attending colorectal surgical services for constipation will be eligible for recruitment and assessed against the eligibility criteria. Such patients will mainly include referrals from secondary care. These will be identified and invited for eligibility assessment by outpatient teams. Some patients may have progressed through the earlier CapaCiTY01 and/or CapaCiTY02.

#### Inclusion criteria

The inclusion criteria are:

- Age 18–70 years
- Patient self-reported problematic constipation
- Symptom onset more than 6 months prior to recruitment
- Symptoms that meet the American College of Gastroenterology definition of constipation [82]
- Constipation that has not responded to treatment to a minimum basic standard (NHS Map of Medicine 2012 (http://mapofmedicine.com/care-pathway-content-update-publication/)), lifestyle and dietary measures and ≥2 laxatives or prokinetics tried (no time requirement) (see Additional file 4)
- Ability to understand written and spoken English (due to questionnaire validity).
- Ability and willingness to give informed consent
- Failure of non-surgical interventions (minimum of nurse-led behavioural therapy).
- Internal rectal prolapse as determined by clinical examination and INVEST, fulfilling the two following diagnostic criteria

1. Intra-anal or intra-rectal intussusception with/without other dynamic pelvic-floor abnormalities (e.g. rectocele, enterocele, perineal descent)
2. Deemed to be obstructing on defecating proctogram, i.e. trapping contrast and/or associated with protracted or incomplete contrast evacuation using normal ranges [54] (by expert review)

#### Exclusion criteria

The study interventions necessitate the exclusion of major causes of secondary constipation. In detail:

- Significant organic colonic disease (red-flag symptoms, e.g. rectal bleeding previously investigated); inflammatory bowel diseases; megacolon or megarectum (if diagnosed beforehand (the study will provide a useful estimate of the prevalence of such cases in referral practice)); severe diverticulosis/stricture/birth defects deemed to contribute to symptoms (incidental diverticulosis if known is not an exclusion)
- Major colorectal resectional surgery
- Current overt pelvic organ prolapse (bladder, uterus) or disease requiring obvious surgical intervention other than LVMR
- Previous rectopexy
- Sacral nerve stimulation (SNS) *in situ*
- Rectal impaction (as defined by digital and abdominal examination: these form part of the NHS Map of Medicine basic standard) (http://mapofmedicine.com/care-pathway-content-update-publication/)
- Significant neurological disease deemed to be causative, e.g. Parkinson’s, spinal injury, multiple sclerosis, diabetic neuropathy (not uncomplicated diabetes alone)
- Significant connective tissue disease, e.g. scleroderma, systemic sclerosis and systemic lupus erythematosus (not hypermobility alone)
- Significant medical comorbidities and activity of daily living impairment (based on Barthel index ≤11 in apparently frail patients)
- Major active psychiatric diagnosis, e.g. schizophrenia, major depressive illness and mania
- Chronic regular opioid use (at least once daily use), where this is deemed to be the cause of constipation based on temporal association of symptoms with onset of therapy
- Pregnancy or intention to become pregnant during study period
- Known severe intra-abdominal adhesions

### Study procedures

#### INVEST radio-physiological investigations

Participants will have undergone standard (clinically routine) investigations to determine eligibility for surgery. However, some patients may have missed specific tests that are required to meet the INVEST standard of the overall programme (or not had tests conducted in last 12 months). In such cases, individual missing investigations will be performed to meet the standard below, with the exception of whole-gut transit studies. In order to avoid unnecessary repeated radiation, whole-gut transit studies performed in the last 12 months (even if using a different marker protocol) may be carried forward if a clear diagnosis of either delayed or normal whole-gut transit time has been confirmed.

Routine NHS practice (10-day NHS rule) will apply in respect of women between menarche and menopause. Participants who may potentially be pregnant will have a serum or urine pregnancy test performed as per routine care.

INVEST includes:

- Anorectal manometry using high-resolution methods [83–85] to determine defined abnormalities of rectoanal pressure gradient during simulated evacuation [36, 86, 87].
- Balloon sensory testing using standardised methods [88, 89] (2 ml air per second to maximum 360 ml) to determine volume inflated to first constant sensation, defaecatory desire and maximum tolerated volumes. Rectal hyposensation and hypersensation defined in accord to gender-specific normative data on 91 healthy adults [90]. The rectoanal inhibitory reflex will also be elicited by 50 ml rapid inflation (if necessary in 50 ml aliquots up to 150 ml).
- Fixed volume (50 ml) water-filled rectal balloon expulsion test [36, 86, 91, 92] in the seated position on a commode. Abnormal expulsion is defined as abnormal if failure to expel within 1.0 minute of effort for men and 1.5 minutes for women [93].
- Whole-gut transit study using serial (different-shaped) radiopaque markers over 3 days with single plain radiograph at 120 hours [77, 94].
- Fluoroscopic evacuation proctography using rectal installation of barium porridge to defaecatory desire threshold (or maximum 300 ml) and evacuation on a radiolucent commode [54, 83–85, 95] with pre-opacification of the small bowel (for enterocele). Radiation dose, proportion of contrast evacuated and time taken will be recorded, as well as “functional” (i.e. pelvic-floor dyssynergia) and “structural” features deemed obstructive to defaecation (e.g. rectocele, enterocele and intussusception) [36, 90].

Participants will be given the results of investigations by the physiologist or radiologist.

#### Laboratory assessments

Serum or urine pregnancy testing will be performed by local NHS biochemistry laboratories as per standard NHS policy prior to radiological and surgical procedures.

#### Pelvic-floor MDT confirmation

As part of the whole CapaCiTY programme, a national MDT has been convened to develop a standard set of criteria for surgical eligibility to be used by local MDTs. These criteria have been coalesced into a trial case report form (CRF) that will be used to validate eligibility for each patient before randomisation.

#### Randomisation procedures

Randomisation will be delivered following recruitment (after full eligibility and all baseline assessments). Randomisation will be stratified by sex and female participants further stratified by centre. The Pragmatic Clinical Trials Unit (PCTU) has developed a validated online randomisation system, which will be accessed by suitably trained and delegated researchers at recruiting sites and will follow the PCTU approved standard operating procedure (SOP) for the study.

#### Blinding

Patients and clinicians are necessarily aware of allocation to different waiting times. For quantitative analysis, an analysis plan will be developed and signed off by investigators and statisticians who are blind to allocation status and index intervention. No quantitative analysis will be undertaken until the analysis plan is signed off.

### Study interventions

#### Laparoscopic ventral mesh rectopexy (LVMR) (see Additional file 5)

Participants will attend for surgery at their allocated time with admissions procedures as per routine clinical care with normal preparation, e.g. bowel cleansing. Perioperative care will proceed with normal adjuncts (informed NHS consent, World Health Organization (WHO) surgical checklist, appropriate broad spectrum antibiotic prophylaxis, venous thromboembolism (VTE) prevention, patient warming and urinary catheter insertion). Surgery can be performed as a day-case procedure within an enhanced recovery programme [96], although most patients will have an overnight stay. Consent will include discussion of the risks of conversion to open surgery and specific complications listed below. A phosphate enema or similar (optional) may be used to clear the rectum.

The exact surgical technique will be surgeon-specific (based on individual preference) but in accord with expert guidance [73] and training. All participating surgeons will require sign-off by a delegated surgical team provided by the Pelvic Floor Section of the Association of Coloproctology. Where required, preceptorship will be provided to meet sign-off requirements (at the time of writing, all participating surgeons are experts in this technique).

In brief, after positioning the patient (modified lithotomy position on a non-slip mat) and port-site insertion (using standard equipment and technique), the rectosigmoid junction is retracted to the left and a peritoneal incision is made over the right side of the sacral promontory and extended in an inverted J-form along the rectum and over the deepest part of the pouch of Douglas. Special care is taken not to damage the right hypogastric nerve. Denonvillier’s fascia is incised and (in women) the rectovaginal septum is broadly opened. Limited rectal mobilization and lateral dissection is performed as required to expose the distal rectum and pelvic floor. A strip of trimmed mesh (biologic or synthetic) is inserted. Using non-absorbable or slowly absorbable sutures (polydioxanone (PDS) is recommended), the mesh is sutured to the ventral aspect of the distal rectum and further fixed to the lateral seromuscular borders of the rectum proximal and distal to the incised pouch of Douglas +/- pelvic floor. The mesh is fixed upon the sacral promontory using either sutures or an endofascia stapler. Limited traction is exerted on the rectum as required to obliterate the intussusception +/- rectocele. If deemed necessary, the posterior vaginal fornix may be elevated and sutured to the anterior aspect of the mesh; this allows closure of the rectovaginal septum and correction of a mid-compartment prolapse, if present. The lateral borders of the incised peritoneum are then closed over the mesh. This elevates the new pouch of Douglas over the colpopexy and completely covers the mesh with peritoneum. No drain is usually required. Ports should be closed directly (endoclose for lateral ports) owing to the high risk of early and late port site hernias in this group of patients with potential connective tissue laxity.

Post-operative management will be as per routine clinical care. This is usually an overnight hospital stay followed by urinary catheter removal, mobilisation and discharge. Post-operative laxatives use is standardised to a weaning course of Movicol/Laxido three times daily (TDS) immediately post-operative for 1 day, then reduced according to ease of bowel movements. Medication will be post-operatively recorded on a drug chart by the anaesthetists. This prevents post-operative constipation from immobility, narcotics and general anaesthesia, which if left untreated may cause painful straining on the mesh and thus protracts in the sacral promontory periosteum, potentially leading to readmission. The surgeon should aim to discharge patients 1 day post-operative. Length of stay will, however, be determined by clinical evaluation and may be longer if required. Quality control of LVMR procedures will be conducted according to expert panel review, as per the relative SOP.

#### LVMR 30-day follow up

Clinical recurrence of rectal prolapse will be determined based on physical examination. Morbidity and mortality data will be collected, in addition to treatment of any complications arising from LVMR surgery. The 30-day readmission rates will also be recorded. A CRF will be used to capture intra-operative and post-operative data (see surgery-specific outcomes).

#### Concomitant medications

It is inevitable that participants will seek recourse to laxatives and other dietary supplements during the course of the programme. Experience shows that complete prohibition can lead to unreported laxative use, which might confound findings. Although we will strongly discourage *ad libitum* medication usage and specify a defined breakthrough regimen, we will record co-treatment with sufficient fidelity and integrity to enable use as covariates in analyses using a specific diary for this purpose. A concomitant medications list including a shortlist of contributory or confounding medications will be used for filtering on data entry.

### Schedule of assessment (see Fig. 1)

**Fig. 1:**
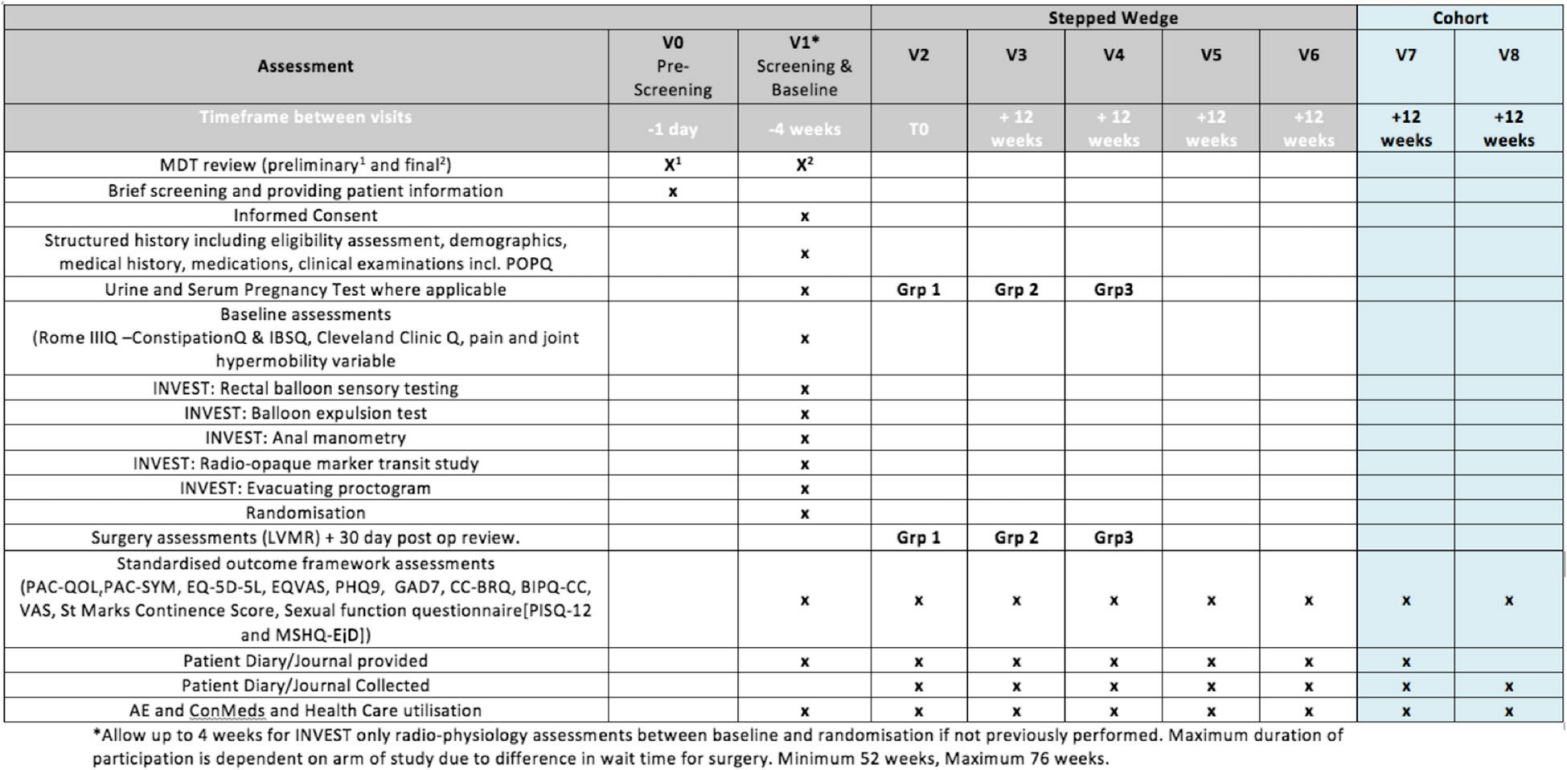
Standard Protocol Items: Recommendations for Interventional Trials (SPIRIT) diagram. *MDT* Multidisciplinary Decision Team, *IBSQ* Irritable Bowel Syndrome Questionnaire, *LVMR* Laparoscopic Ventral Mesh Rectopexy, *PAC-QOL* Patient Assessment of Constipation Quality of Life questionnaire, *PAC-SYM* Patient Assessment of Constipation Symptoms Questionnaire, *EQ-5D* EuroQol Health Outcome measure, *EQVAS* EuroQol visual analogue scale, *PHQ-9* Patient Health Questionnaire -9, *GAD7* Generalized Anxiety Disorder Questionnaire, *CC* chronic constipation, *BIPQ* Brief Illness Perception Questionnaire, *AE* adverse events, *ConMeds* concomitant medications, *V* visit, * POPQ* Pelvic Organ Prolapse Quantification System, *Rome IIIQ * constipation questionnaire based on the Rome III criteria, *CC-BRQ* Behavioural Response to illness Questionnaire

#### Visit 0 - Pre-screening: eligibility assessment

A suitably trained and delegated local researcher will screen for basic eligibility within outpatient clinics or by phone (or later face-to-face interview based on patient choice) on the basis of a simplified inclusion/exclusion criteria proforma, and listed for LVMR (in some cases/NHS settings based on preliminary MDT review). Participants will be recorded on a screening log and each will be allocated a sequential study number. Eligible participants will be provided with adequate explanation of the aims, methods, anticipated benefits and risks of the study and will take away or be posted an invitation letter and patient information sheet (PIS). Patients will be given at least 24 hours to consider participation.

The study screening number will be allocated as follows:

- Study code 03
- Site code – 3-letter code for each site
- Participant code – 4-digit code given consecutively and attributed at each site

For example, the first participant screened at Bart’s Health Trust would be assigned the code 03-BLT-0001. If they were then recruited to the study, they would retain this number.

#### Visit 1 - consent, screening, and baseline assessments

Visit 1 will be conducted face-to-face in the clinic or private research environment. Following a detailed discussion about the trial and review of the PIS, patients who are basically eligible and who agree to participate will complete written informed consent, followed by screening and confirmation of eligibility for randomisation by obtaining structured medical and surgical history and review of findings on physical examination. Thereafter, additional baseline outcome assessments will be conducted. These include several key validated assessments that profile patients for important characteristics informing disease pathophysiology and important potential predictors of treatment response. All have been selected on the basis of trade-off between adequate detail and achievable brevity. These instruments will be coalesced into a single booklet (he design and presentation have been optimised by patient representatives).

##### Screening/confirmation of eligibility

Screening and confirmation of eligibility will be performed as follows:

- Structured medical and surgical history obtained by interview, including medication usage
- Clinical examination findings (carried forward if performed previously within the last 3 months): standardised examination of the perineum/anus/rectum/vagina including POP-Q assessment of rectocele
- Review of clinical radio-physiological investigations (some further tests may be required to meet INVEST standard)
- Final review by pelvic floor MDT (as NHS England draft recommendation) to confirm appropriateness for surgery

##### Standardised outcome framework

The standardized outcome framework will comprise:

- PAC-QOL, PAC-SYM, EQ-5D-5L and EQVAS, PHQ9, GAD7, CC-BRQ, BIPQ-CC, St Marks Incontinence score, PISQ-12 in women and MSHQ-EjD Short Form in men)
- Baseline 2-week patient bowel diary and health economics journal will be given

##### Other baseline-only assessments

Other baseline-only assessments comprise:

- Constipation (2006) and IBS (2006) modules of the Rome III questionnaire
- Cleveland Clinic constipation questionnaire [97]
- Brief, chronic pain, autonomic and joint hypermobility assessments

Randomisation will be performed only once full eligibility has been confirmed and all baseline assessments taken (which may require INVEST). Urinary pregnancy testing will be made available to women of child-bearing potential at eligibility assessment and advice will be given to all women on the need to prevent pregnancy during the study intervention period.

#### Visit 2 - run-in for surgical interventions

Participants will be randomized (at visit 1) to three arms with different lengths of delay before surgery. In all arms there will be a period of 4 weeks post-eligibility to arrange the logistics of surgery (T–4w to T0) and ensure that patients have returned to their normal life routine after various assessments. Subsequently, LVMR will be performed at T0 in group I (visit 2); T12 (12 weeks) in group II (visit 3) and T24 (24 weeks) in group III (visit 4).

#### Visit 2–8 - follow-up outcome assessments

All patients will complete the standardised outcome framework (inclusive of PAC-QOL and PAC-SYM) questionnaires at T–4, T0 and 12, 24, 36, 48, 60 and 72 weeks post run-in (see Additional file 2). This ensures that 24-week and 48-week post-surgery follow-up data on the primary and secondary outcomes are collected in all patients, whilst maintaining blinding of group allocation. Thereafter, participants will leave the study and return to “routine clinical care” as determined within their local NHS institution. During the first 24 weeks post-surgery patients will be quarantined from further intervention, excepting emergency interventions (e.g. for complications).

#### Participant withdrawal (including data collection/retention for withdrawn participants)

Individual participants will be able to drop out at any time during either the treatment or follow up. Data will be retained for intent-to-treat analysis from all participants after the point of consent and recruitment as outlined in the PIS:

- Withdrawal from treatment criteria (no further interventions but follow up data collected){ participants will be withdrawn from the study interventions if they develop any of the following exclusion criteria:
  Becomes pregnant or intends to become pregnant (only in baseline and intervention phases);
  Subsequently diagnosed with proven cause of secondary constipation e.g. Parkinson’s disease or bowel obstruction;
  Develops significant inter-current illness precluding participation;
  Develops acute psychological problem causing safety concern;
  Elective withdrawal.
- Loss to follow up (no further interventions or follow up data collected): participants may be withdrawn from the trial if:
  They become lost to follow up (LTF) after at least three failed attempts by research staff to make contact via two different methods (e.g. phone and letter);
  Participant choses to withdraw and does not wish to participate in follow-up data collection;
  Participant dies or has significant incapacity making follow-up data collection impossible.

#### End-of-study definition

The end of study is defined as the last patient last visit (LPLV). The sponsor, Research Ethics Committee (REC) and local Research and Development (R&D) departments will be informed of the end of study and site closure and archiving procedures will be initiated.

#### Criteria for early termination

If the Data Monitoring and Ethics Committee (DMEC), PSC, REC or sponsor determine it is within the best interests of the participants or trial to terminate the study, written notification will be given to the CI. This may be due to, but not limited to; serious safety concerns, serious breaches, acts of fraud, critical findings or persistent non-compliance that negatively affects patient safety or data integrity. If the study is terminated, participants will be returned to the NHS normal follow up and routine care.

### Data management

The data collected for the trial will be a mixture of routinely collected data, verifiable against the medical records and patient-reported outcome measures (PROMs) or questionnaire data, collected directly in the CRF.

Each recruiting site will be required to keep accurate and verifiable source notes in the medical record relevant to each study participant’s inclusion and continued participation in the study. Data will be collected, transferred and stored in accordance with Good Clinical Practice (GCP) guidelines and data protection requirements. The PCTU SOPs and study data management plan will define the exact process of data collection, transfer and storage and control of study data.

All patient identifiable data, such as consent forms, screening and identification logs will be stored in the investigator site files in secure locked cabinets and/or offices, accessible only to delegated members of the study team. Secure methods of data transfer will be used to return CRFs to the coordinating site for centralized data entry, monitoring, quality control and compliance. A copy of the CRF will be held at the site in accordance with GCP.

A secure online “OpenClinica” trial database will be provided by the PCTU to enable remote data entry of CRFs at sites where this is feasible. This database will provide built-in data validation checks with quality control checks performed by checking a predefined percentage of CRF data against data entered into the database. In addition, on-site monitoring will enable source document verification of records.

The full data set will be collected face to face wherever possible to maximise completeness of data. However, to minimise bias, where possible, a blinded researcher will collect outcome data. Alternatively, the participant will enter outcome data directly into the e-CRF portal for patient-reported outcomes (REDCAP). An automated e-mail reminder will be sent to participants to remind them to complete the questionnaires and diaries every 12 weeks. Telephone or postal follow up will be permitted if necessary. At least three attempts via two different methods (e.g. phone and letter) will be made by research staff to make contact and collect follow-up data, after which the participant may be considered LTF if appropriate (see criteria for withdrawal).

#### Confidentiality

Information related to participants will be kept confidential and managed in accordance with the Data Protection Act, NHS Caldecott Principles, The Research Governance Framework for Health and Social Care and the conditions of REC approval.

Identifiable information to be collected from the participants includes full name, date of birth (DOB), hospital number and contact details at screening. This information will be used to contact participants but will not leave the study site without prior consent. All CRFs will be pseudonymised. The participant’s GP will be informed of their participation in the study, but they may opt out at the time of consent.

The trial data will be made available to suitably qualified members of the research team, study monitors and auditors, the REC and regulatory authorities as far as required by law. The participants will not by identifiable with regards to any future publications relating to this study.

#### Record retention and archiving

When the research trial is complete, it is a requirement of the Research Governance Framework and Sponsor Policy that the records are kept for a further 20 years. For trials involving Barts Health Trust patients, undertaken by Trust staff, or sponsored by BH or Queen Mary, University of London (QMUL), the approved repository for long-term storage of local records is the Trust Modern Records Centre.

Each site will be required to archive local site files and patient identifiable information such as consent forms and screening logs for a period of 20 years. At the end of the 20 year retention period, the Records Management team will alert R&D that the records are due for disposal. The chief investigator (CI) and sponsor will be informed and the full agreement of everyone concerned will be obtained before any records are destroyed.

### Statistical considerations

#### Sample size

The sample size has been calculated using the primary clinical outcome, a change in mean PAC-QOL score [74]. This widely used, psychometrically robust measure of overall treatment response with concurrent validity for patient global ratings of success has been used in previous trials of behavioural therapies and surgical trials [52] (including trials of LVMR) in CC [75]. For a chronic condition such as CC, a difference of 1.0 point in the primary outcome (score range = 1–4) can be considered clinically important and also the notional minimum required to justify the cost and invasive nature of LVMR, or of a more complex and expensive treatment.

Previous trials have shown a 1-point decrease in PAC-QOL from pre-operative to 48 weeks (1 year) post-surgery [52]. Using a stepped-wedge design, we hypothesize that PAC-QOL score at any time point during follow up will be approximately 1.0 point lower than in participants pre-operatively.

Sample size was calculated by simulation using the simsam package in Stata [98]. We assumed PAC-QOL follows a normal distribution over all time points with a standard deviation of 1.5 and with correlation between repeated assessments equal to 0.5.

Simulation shows that detection of a 1.0-point difference in 6-month PAC-QOL, with 95% power (purposely chosen to reflect the magnitude and risk of the intervention) at the 5% significance level, requires 34 participants in each of the three arms. Allowing for a 10% loss to follow up, a sample size of 38 is needed per arm (i.e. a total sample size of 114 patients across the three arms). Should the correlation between repeated assessments be lower than 0.5, a sample size of 114 will still provide at least 90% power for the study. This was calculated using the same simulation procedure with correlations of 0.3 and 0.1.

### Methods of analysis

#### Clinical outcomes

##### Primary objective

Primary outcome

PAC-QOL scores at the time-points T0, T12, T24, T36 and T48 weeks in the three arms will be analysed using a mixed linear regression model, with random effects for participants and a fixed effect of time since randomisation (potentially considering a random effect for time as well to relax the assumption of same time trend for each participant) to estimate mean differences between PAC-QOL score before and after LVMR. The comparison of primary interest is between the score at 24 weeks after surgery and the score at baseline. Missing data will be imputed through multiple imputation by chained equations.

Secondary outcome

PAC-SYM scores will be analysed by the same approach as described above.

##### Secondary objectives

All clinical outcomes derived from the standardised outcome framework will be analysed at 0, 24, 48 and potentially 60 and 72 weeks post-operatively. Outcomes will take the form of count (change in number of symptom episodes), ordinal (patient’s global impression of success) and continuous (questionnaire scores) data. Mixed models appropriate to the outcome data types will be fitted to estimate the treatment effect, adjusting for baseline values, gender, and breakthrough medication use as a potential confounder.

All participants randomized to the three groups will be analysed according to their allocation: we would allow for +/- 2 weeks from the scheduled intervention date. Eventual deviations from this time buffer will be taken into account by a modified intention-to-treat analysis. Analysis will be performed using proprietary software (Stata, Stata Corp. TX, USA) with *p* < 0.05 taken to indicate statistical significance.

### Health economic outcomes

Within-trial stochastic analysis will compare the cost/success and cost/quality-adjusted life year (QALY) of LVMR. Patient-level cost-effectiveness will be analysed using standard bootstrapping methods to generate cost-effectiveness acceptability curves exploring value for money.

Cost-effectiveness models that extrapolate beyond 3–6 months duration are problematic in adult constipation, as subsequent care and outcomes are contingent upon subsequent care received and the underlying disease process. However, the CapaCiTY programme as a whole provides a unique opportunity to construct probabilistic models exploring optimal pathways from effectiveness and cost-effectiveness perspectives.

Since patients will (within the CapaCiTY programme) be followed along a pathway that includes a series of steps of care, it will be possible to construct costs and outcomes for a range of patient pathways providing comparative longer-term cost-effectiveness estimates. For example, it will be possible to ask whether INVEST or no-INVEST-led first-line care leads to lower overall costs or improved outcomes. Patient-level data from recruitment through the various work packages will be used to construct pragmatic, probabilistic models to explore optimal pathways from effectiveness and cost-effectiveness perspectives.

Analyses from NHS and societal perspectives will be supported by recording relevant resource use during each work package, and a common panel of outcomes. Adjustment for time preference will be at the socially accepted rate for cost-effectiveness analyses (currently 3.5% for costs and benefits).

### Qualitative interviews

Interviews will be digitally recorded, anonymised, transcribed verbatim and analysed using a pragmatic thematic analysis and NVivo8 software (QSR International Ltd, Warrington, UK) for data management. Data analysis will be developed as outlined by Fereday and Muir-Cochrane (1997) in the first instance by mapping key concepts derived from the transcripts (“charting”) and extracting emergent themes from the transcripts. Independent analyses will be conducted and resulting codes and themes will be compared and refined in discussion. Emergent themes, together with captured observational data, will form the basis of analytical interpretation.

## Ethical considerations

### General

The study will be carried out in accordance with the ethical principles in the Research Governance Framework for Health and Social Care, Second Edition, 2005 and its subsequent amendments and applicable legal and regulatory requirements.

CapaCiTY study 3 is the last of the three trials in the CapaCiTY Programme, all of which have been reviewed by the London – City and East REC. Within the programme, the three studies have separate protocols and patient information sheets to be consented separately as if they were distinct entities. This is necessary to limit patient information, which would otherwise be over-burdensome. We have discussed the use of sequential consent forms within one pragmatic enriched design with Dr Art Tucker, national ethics advisor and Chair of the London – City and East REC, which confirms this will be practicable.

### Specific

The protocol has been reviewed by Professor Richard Ashcroft, Professor of Medical Ethics and Law at QMUL. Important considerations that have informed pragmatic design include:

- Wait-list controlled design: a waiting list control group serves the purpose of providing an untreated comparison for the active treatment group, while at the same time allowing the wait-listed participants an opportunity to obtain the intervention at a later date. In keeping with the basic ethical tenets of this design [99], the average wait will be shorter than that for routine services. This is achieved by randomising patients to receive urgent (4 weeks) or routine intervention as opposed to all having routine status as would be normal NHS clinical care. Current waiting times at most included centres are approximately 3–6 months for surgery, whereas the mean waiting time in the study will be 3 months. Survey evidence from 100 patients indicates that for a chronic condition such as CC, patients are prepared to accept a randomization strategy that allocates them to a one in three chance of waiting up to 24 weeks for surgical treatment;
- Limitation of intimate examinations to one time point (not repeated if performed before recruitment);
- Timings of outcomes: within the standardised outcome framework, outcomes will be undertaken at fixed intervals of 12 weeks before and after the intervention to 48 weeks follow up within the stepped-wedge study and thereafter in 12-week intervals within the cohort assessments up to 72 weeks. For a period of 24 weeks of follow up post-surgery, patients will not progress to further therapies, thus preventing outcome “contamination”. This “quarantine” period from major therapy progression is required to give a reasonable clinical impression of outcome. This delay is akin to that in usual NHS care during which general supportive care will be provided while further interventions are considered. Thus, this proposed “quarantine” period to 6 months confers no disadvantage and may even represent an acceleration of treatment progression. Ethically, this is viewed as a reasonable trade-off for the commitment to the research programme;
- Recruitment and consent: study 3 represents one of the three studies incorporated in the NIHR-funded CapaCiTY programme. Although patients may have moved sequentially through earlier treatments (and therefore studies) during the programme course, study 3 will be consented as a distinct single entity.

The investigating team has no conflicts of interest.

## Safety considerations

### Surgery

LVMR has a number of established specific complications in addition to the general risks of surgery. Data on these complications are in the public domain [72] and can be considered to be expected events. These will, however, still be recorded for outcome reporting.

#### Intra-operative complications

Intra-operative complications include inadvertent injury to other intraperitoneal viscera. These are common to all types of laparoscopic surgery, such as:

- Bowel, ureter, bladder or vaginal injuries or perforations
- Vascular or nerve damage

#### Post-operative complications

- Urinary retention (<10%)
- Urinary tract infection
- Worsening of, or *de novo* urinary incontinence
- Port site complications (early or late port site hernia; bleeding or wound infection)
- Pelvic sepsis
- Pelvic pain
- Haemorrhage especially from the posterior vaginal wall
- Vaginal or rectal perforation
- Faecal impaction (rare)
- Small bowel obstruction
- Sexual dysfunction (rare)
- Dyspareunia (uncommon) – usually resolves with time [100]
- Osteomyelitis of the sacrum and spondylodiscitis [101]
- Venous thromboembolism

#### Prosthesis-related complications

- Minor mesh complications
- Mesh infection (<3%)
- Mesh erosion (<3%)
- Mesh sinus

Minor mesh complications can be managed by local measures including suture sinus removal, mesh trimming, performed endo-rectally or endo-vaginally with subsequent healing. Major mesh complications include (1) generalised mesh sepsis requiring mesh removal endo-rectally or trans-abdominally or both, with or without partial or complete rectal excision and (2) rectovaginal fistula also requiring mesh removal endo-rectally or trans-abdominally or both +/-partial or complete rectal excision.

### INVEST safety considerations

Patients undergoing INVEST-guided therapy will have two radiological procedures (whole-gut transit study and evacuation proctography) using ionising radiation as outlined above. The combined dose of these procedures (~1.2mSv) is equivalent to less than 7 months annual background radiation dose from living in the UK (this has been recertified by Barts Health NHS Clinical Physics Department based on doses from 20 equivalent procedures). Further, these investigations would be carried out in routine clinical practice in many centres for patients at the same point as recruitment to this study.

### Insurance and indemnity

In the event that something does go wrong and patients are harmed during the research and this is due to someone’s negligence then they may have grounds for legal action against the sponsor QMUL, but they may have to pay their legal costs. Insurance and indemnity is provided by the sponsor.

### Safety reporting

Serious adverse events (SAEs) that are considered to be “related” and “unexpected” are to be reported to the sponsor within 24 hours of learning of the event and to the REC within 15 days in line with the required timeframe. The CI will send the Annual Progress Report to the REC and to the sponsor.

#### Expected SAEs

The following SAEs are expected to occur rarely in this patient population and will not be reported:

- Hospital admission for exacerbation of constipation symptoms including impaction
- Hospital admission for unrelated elective surgical procedures or accidental injury
- Prolongation of hospitalisation due to complications from surgery

#### Urgent safety measures

The CI may take urgent safety measures to ensure the safety and protection of the clinical trial subjects from any immediate hazard to their health and safety. The measures should be taken immediately. In this instance, the approval of the REC prior to implementing these safety measures is not required. However, it is the responsibility of the CI to inform the sponsor and Main Research Ethics Committee (via telephone) of this event immediately.

The CI has an obligation to inform both the REC in writing within 3 days, in the form of a substantial amendment. The sponsor (Joint Research Management Office (JRMO) for QMUL) must be sent a copy of the correspondence with regards to this matter.

#### Overview of the safety reporting responsibilities

The CI/PI has overall responsibility for oversight of pharmacovigilance. The CI/PI has a duty to ensure that safety monitoring and reporting is conducted in accordance with the sponsor’s requirements (see Additional file 6).

## Monitoring and auditing

### Risk assessment

The PCTU quality assurance manager will conduct a study risk assessment in collaboration with the CI. Based on the risk assessment, an appropriate study monitoring and auditing plan will be produced according to PCTU SOPs. This monitoring plan will be authorised by the sponsor before implementation. Any changes to the monitoring plan must be agreed by the PCTU QA manager and the sponsor.

A study may be identified for audit by any method listed below:

- A project may be identified via the risk assessment process;
- An individual investigator or department may request an audit;
- A project may be identified via an allegation of research misconduct or fraud or a suspected breach of regulations;
- Projects may be selected at random. The Department of Health states that Trusts should be auditing a minimum of 10% of all research projects;
- Projects may be randomly selected for audit by an external organisation.

Internal audits may be conducted by a sponsor’s or funder’s representative.

### Quality assessment of LVMR

Monitoring and quality control will be conducted remotely via video submission and assessed against the standardised LVMR protocol and assessment criteria (see Additional file 7). Monitoring will take the form of planned, random and triggered sessions.

#### Planned monitoring

All PIs must record and submit the unedited and anonymised video of the LVMR performed in the first patient enrolled in the CapaCiTY study 3. Each video will be allocated to two peer reviewers of a three-member expert panel. Based on blinded assessment of unedited and anonymised videos by expert review, the panel will decide whether the PI is “adherent” to the standardized technique. Any disagreement will be solved by consensus after consulting a third independent expert. If deemed “non-adherent” to the standardized technique, the site will be notified that a step needs to be corrected. The PI must submit the unedited and anonymised video of the LVMR performed in the second patient enrolled in the CapaCiTY study 3. In case of “failure” to comply with the standardized surgical technique for LVMR or following a second judgment of “non-adherence” to the standardized technique, this will trigger an on-site training and monitoring session for the site. Monitoring will continue until adherence is achieved. Otherwise a third “non-adherence” or second “failure” judgement will result in withdrawal of the site/PI from the study.

#### Random monitoring

All PIs must record and submit the unedited and anonymised video of the LVMR performed in a randomly selected patient enrolled in the CapaCiTY study 3 (one in five at site level). The adherence to the standardized technique will be established by consensus as described for the planned monitoring.

#### Triggered monitoring

The DMC will review the morbidity and mortality rates, adverse and serious adverse events from all sites. Safety concerns may trigger additional monitoring or on-site training and mentorship visits by an expert panel. Repeated “non-adherence” or “failure” to comply will result in PI and site withdrawal.

## Devices and licenses

### Devices

The following is a list of all devices used. None are specific to the research itself and all are currently used in routine clinical practice. All are European-conformity (CE)-marked and approved for use in the UK

- Disposable proctoscope (supplier as in local NHS practice). This will be commonly be used as part of clinical examination at baseline and is also used to introduce barium paste into the rectum during INVEST;
- High-resolution anorectal manometry catheters and rectal balloons for anal manometry/rectal sensory testing: various suppliers (part of INVEST – see above);
- Balloon catheters for the balloon expulsion test (part of INVEST – see above);
- Radiopaque markers for colonic transit study: various suppliers (part of INVEST – see above);
- Standard departmental x-ray equipment including the radiolucent commode for the proctogram (part of INVEST- see above);
- Surgical instrumentation including disposable and reusable instruments;
- Mesh:
  Synthetic: titanium-coated lightweight polypropylene;
  Biologic: Strattice, Permacol;
  Mixed: biologic and synthetic;
- Suture material: any; usually long-term absorbable material e.g. PDS.

### Licenses

Most of the questionnaire-based tools are free to use within the public domain. The permissions/licenses to use all instruments will be sought with finance where required:

- PAC-QOL score: MAPI registered
- PAC-SYM score: MAPI registered
- MSHQ-EjD: MAPI registered
- EQ-5D-5L: registered

No costs are associated with the following tools:

- Depression, anxiety and somatisation modules of the Patient Health Questionnaire
- Illness perception questionnaire
- Composite Rome III/Cleveland Clinic constipation questionnaire
- Brief, chronic pain, autonomic and joint hypermobility
- Negative perfectionism
- Avoidant and “all or nothing” behaviour subscales of the behavioural response to illness questionnaire

## Trial management

Each participating centre will identify a site-specific PI who will nominate a local contact for that centre (this may be the PI). The PI and local contact will:

- Be familiar with the trial;
- Liaise with the Programme Management Group (PMG);
- Ensure that all staff involved in the trial are informed about the trial and have received requisite training;
- Ensure that mechanisms for recruitment of eligible participants, including the availability of participant information and data collection tools, are in place;
- Monitor the effectiveness of data collection tools and participant information and discuss the reasons for non-recruitment with relevant staff;
- Ensure site staff collect necessary trial data and perform quality checks;
- Notify the CI of any SAEs and serious breaches within required timelines;
- Make data available for verification, audit and inspection processes as necessary;
- Respond to requests for documentation and data required for centralised monitoring;
- Ensure that the confidentiality of all information about trial participants is respected by all persons.

Site initiation will be conducted with each site. This will include training in the trial protocol and SOPs, such as data collection, randomisation and taking informed consent. Evidence of appropriate training, local approvals and essential documentation will be required before participants are enrolled at each site. Training will be documented on training logs.

### Trial committees

The project will be under the auspices of the CI and the PCTU. The project will be overseen by a Programme Steering Committee (PSC). The composition and responsibilities of the PSC will comply with the NIHR guidance and PCTU SOP on Trial Oversight Committees. The role of the PSC is to provide overall supervision of the study on behalf of the sponsor and funder to ensure the study is conducted in accordance with the principles of Good Clinical Practice (GCP) and relevant regulations.

The responsibilities of the PSC will include:

- Ensuring that views of users and carers are taken into consideration
- Advising on the trial protocol
- Advising on changes in the protocol based on considerations of feasibility and practicability
- Assist in resolving problems brought to it by the PMG
- Monitor the progress of the trial and adherence to the protocol and milestones
- Consider new information of relevance from other sources
- Consider and act on the recommendations of the DMEC, sponsor and/or REC
- Review trial reports and papers for publication

The PSC will meet to review the protocol before the start of the programme and then soon after the first participants are recruited and either meet or teleconference every 6 months thereafter throughout the lifetime of the programme. Representatives of the trial sponsor and funder will be invited to attend.

The PMG will meet monthly initially during study set up and then less frequently, every 2 months. The PMG will be responsible for day-to-day project delivery across participating centres, and will report to the PSC. The PMG will be responsible for monitoring adherence to the study timelines and expected recruitment rates. Regular reports will be produced to enable deviations from the project plan to be identified and contingencies planned, discussed and executed in a timely fashion (see Additional file 8).

The DMEC will be convened. A “Data Monitoring Committees: Lessons, Ethics, Statistics” (DOMACLES) charter will be adopted, and the project team will provide the DMEC with a comprehensive report, the content of which should be agreed in advance by the Chair of the DMEC and follow guidelines set out in the charter. The DMEC will meet at least 4 weeks prior to the PSC to enable recommendations to forwarded.

A constipation research advisory group (CRAG) will be formed as part of a well-developed patient and public involvement (PPI) strategy at QMUL (in close association with the charity Bowel and Cancer Research). This advisory group will comprise eight patients and two lay members from London and Durham. This group will have geographical diversity (north and south) and a disease-appropriate demographic (eight women, two men). The CRAG will review participant information sheets, booklets, diaries and advertising/marketing materials, provide lay representation on the PSC, conduct parallel qualitative analysis, produce lay summaries for dissemination of results, present at local research events and conduct patient focus groups and workshops.

## Discussion

An individual-level stepped-wedge randomised trial serves the purpose of providing an untreated comparison for the active treatment group, while at the same time allowing the wait-listed participants an opportunity to obtain the intervention at a later date. In keeping with the basic ethical tenets of this design, the average waiting time for LVMR (12 weeks) will be shorter than that for routine services (24 weeks). We acknowledge that availability of beds may represent a major bane for this trial. However, we have attempted to overcome this by allowing a 4-week run-in post-eligibility to arrange the logistics of surgery and a 2-week tolerance interval from the scheduled intervention date.

## Trial status

Recruitment is ongoing.

## Additional files

Additional file 1: CapaCiTY programme - design overview with approximate numbers at each stage. (TIF 693 kb)

Additional file 2: CapaCiTY study 3 scheme diagram. (TIF 414 kb)

Additional file 3: Standard Protocol Items: Recommendations for Interventional Trials (SPIRIT) checklist. (DOC 121 kb)

Additional file 4: NHS Map of Medicines – Constipation. (TIF 752 kb)

Additional file 5 Schematic diagram of laparoscopic ventral mesh rectopexy (LVMR). (TIF 166 kb)

Additional file 6: Communication organogram for reporting serious adverse events. (TIF 185 kb)

Additional file 7: Criteria for quality assessment of laparoscopic ventral mesh rectopexy (LVMR). (TIF 498 kb)

Additional file 8: CapaCiTY study 3 Gantt chart. (DOC 49 kb)

## Abbreviations

AE: Adverse event
AR: Adverse reaction
ASR: Annual safety report
BIPQ: Brief Illness Perception Questionnaire
CC: Chronic constipation
CI: Chief Investigator
CPCPH: Centre for Primary Care and Public Health
CRF: Case report form
DMEC: Data Monitoring and Ethics Committee
EC: European Commission
EQ-5D: EuroQol Health Outcome measure
EQ-VAS: EuroQol visual analogue scale
FDD: Functional defaecation disorder
GAD7: Generalized Anxiety Disorder Questionnaire
GAfREC: Governance Arrangements for NHS Research Ethics Committees
GCP: Good Clinical Practice
GP: General Practitioner
HRA: Health Research Authority
IBSQ: Irritable Bowel Syndrome Questionnaire
ICF: Informed consent form
INVEST: Standard panel of radio-physiological tests of colonic and anorectal function
JRMO: Joint Research Management Office
LTF: Lost to follow up
LVMR: Laparoscopic mesh ventral rectopexy
MDT: Multidisciplinary Decision Team
MYMOP2: Measure Yourself Medical Outcome Profile
NHS: National Health Service
NHS R&D: National Health Service Research & Development
NHS REC: National Health Service Research Ethics Committee
NICE: National Institute for Health Care and Excellence
NIHR: National Institute for Health Research
PAC-QOL: Patient Assessment of Constipation Quality of Life questionnaire
PAC-SYM: Patient Assessment of Constipation Symptoms Questionnaire
PCSG: Primary Care Society for Gastroenterology
PCTU: Pragmatic Clinical Trials Unit
PHQ-9: Patient Health Questionnaire -9
PHQ-15: Patient Health Questionnaire-15
PI: Principal Investigator
PIS: Participant information sheet
PMG: Programme Management Group
PPIG: Patient and Public Involvement Group
PRO: Patient-reported outcomes
PSC: Programme Steering Committee
QA: Quality assurance
QC: Quality control
QMUL: Queen Mary, University of London
QOL: Quality of life
RAIR: Rectoanal inhibitory reflex
RCT: Randomised controlled trial
REC: Research Ethics Committee
SAE: Serious adverse event
SAP: Statistical analysis plan
SD: Standard deviation
SDV: Source document verification
SOP: Standard operating procedure
SUSAR: Suspected unexpected serious adverse reaction
VAS: Visual analogue scale
VTE: Venous thromboembolism
WP: Work package
