# Supplementary figures and images for: Stepped-wedge randomised trial of laparoscopic ventral mesh rectopexy in adults with chronic constipation: study protocol for a randomized controlled trial
Source: Trials. 2018 Feb 5;19:90. doi: 10.1186/s13063-018-2456-3 (PMC5800022; doi:10.1186/s13063-018-2456-3)

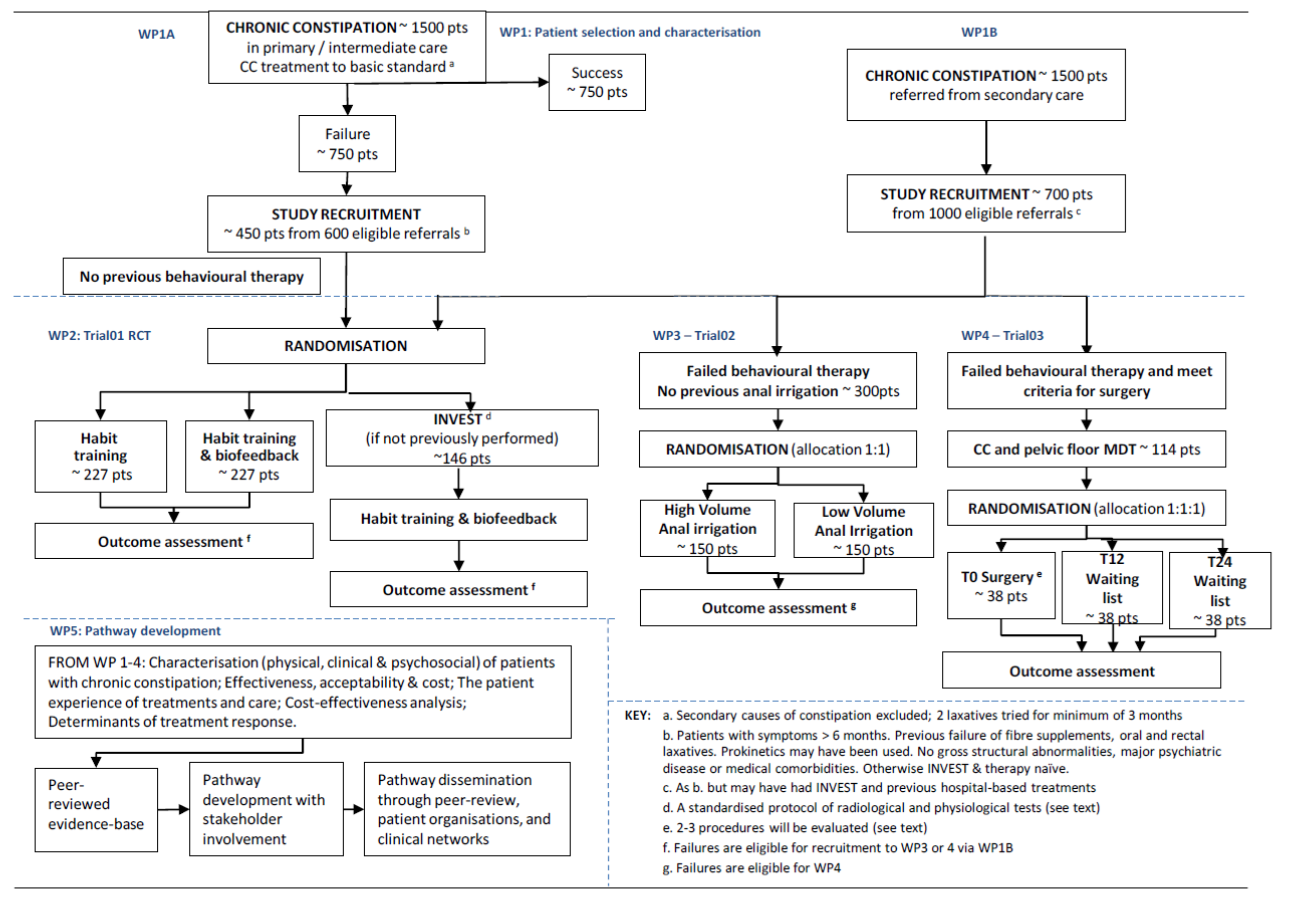

Supplement: Supplementary file 1 — CapaCiTY programme - design overview with approximate numbers at each stage. (TIF 693 kb) [file 13063_2018_2456_MOESM1_ESM.tif]

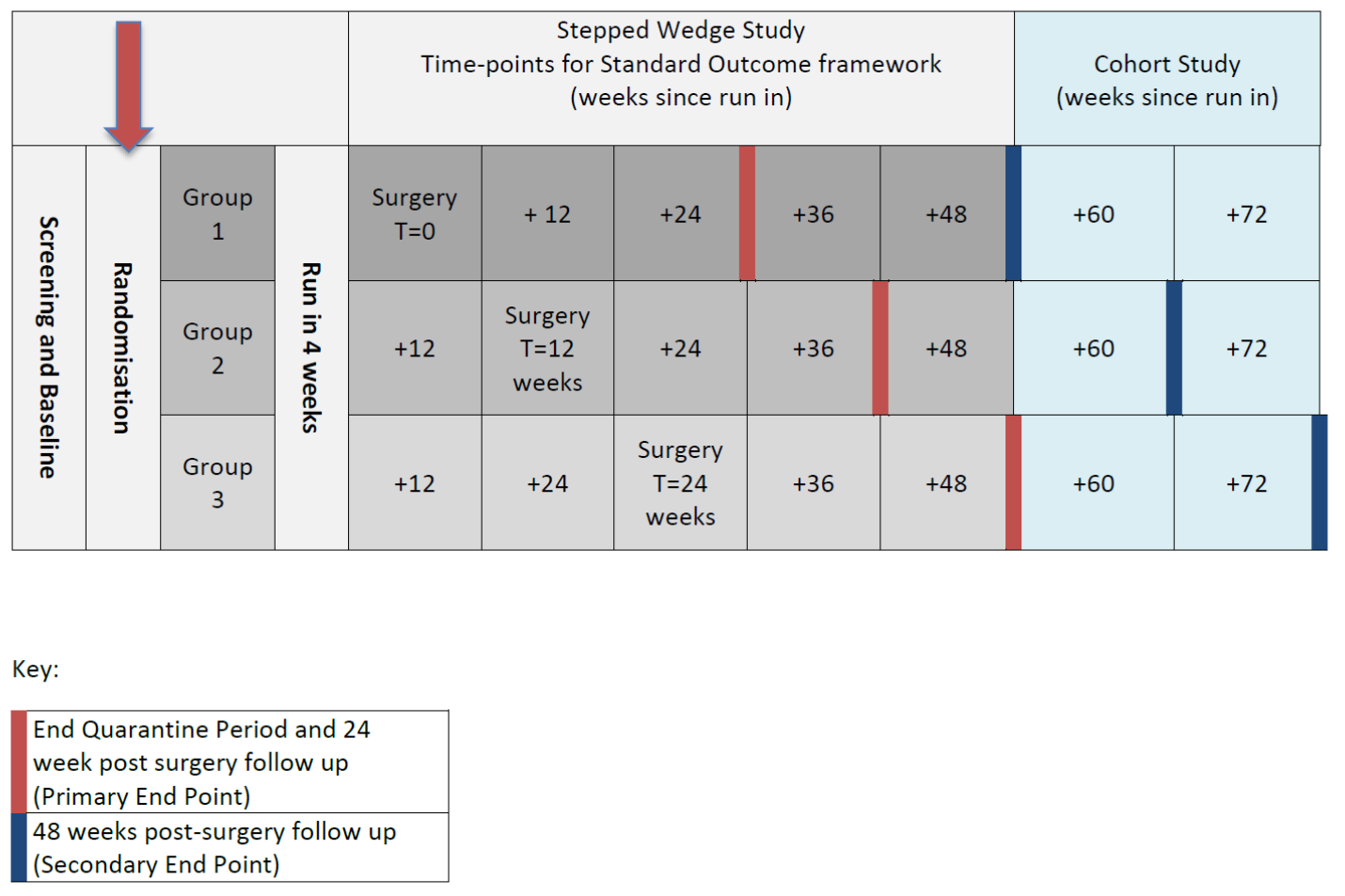

Supplement: Supplementary file 2 — CapaCiTY study 3 scheme diagram. (TIF 414 kb) [file 13063_2018_2456_MOESM2_ESM.tif]

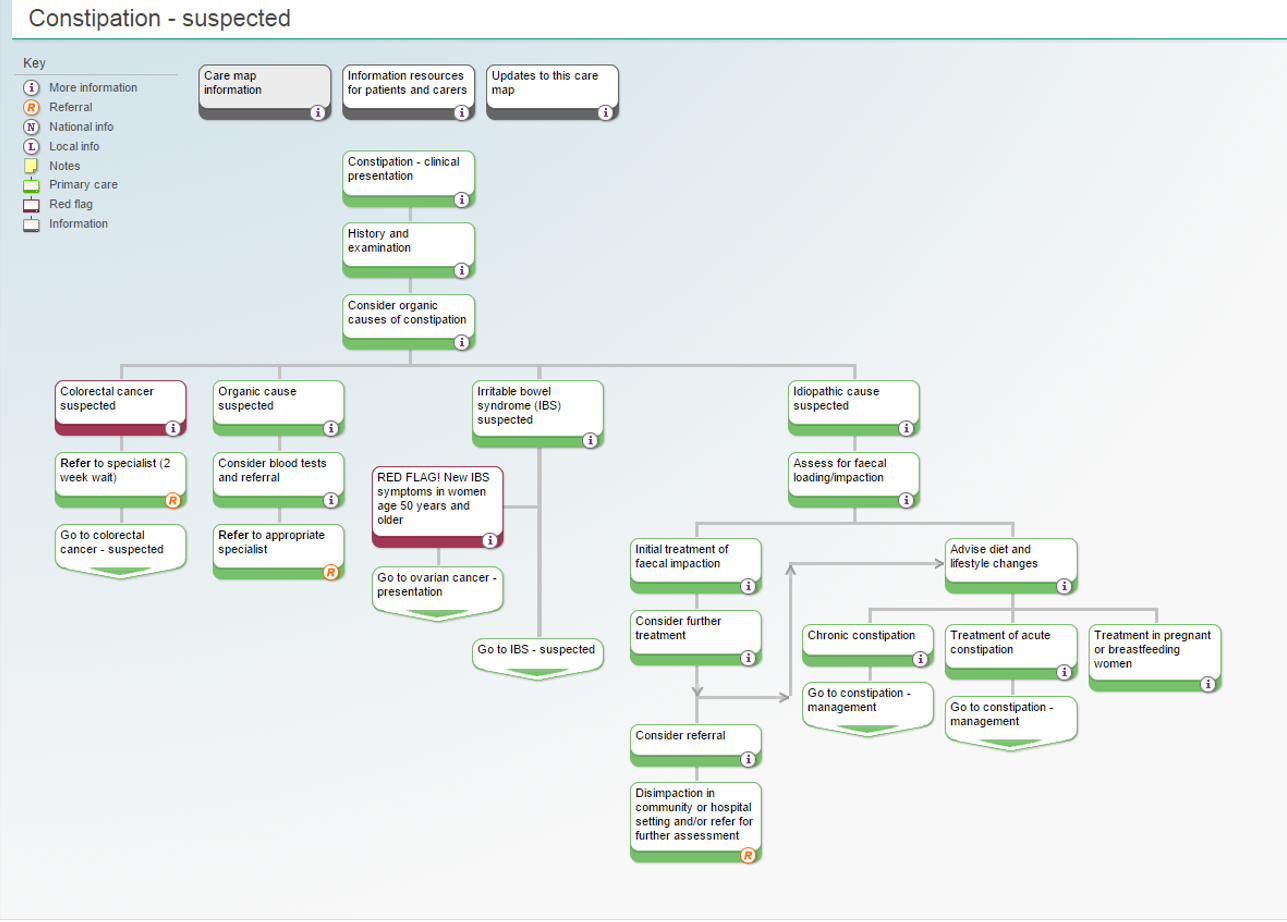

Supplement: Supplementary file 4 — NHS Map of Medicines – Constipation. (TIF 752 kb) [file 13063_2018_2456_MOESM4_ESM.tif]

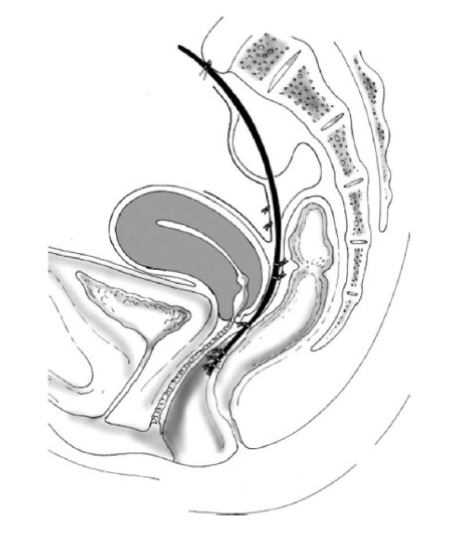

Supplement: Supplementary file 5 — Schematic diagram of laparoscopic ventral mesh rectopexy (LVMR). (TIF 166 kb) [file 13063_2018_2456_MOESM5_ESM.tif]

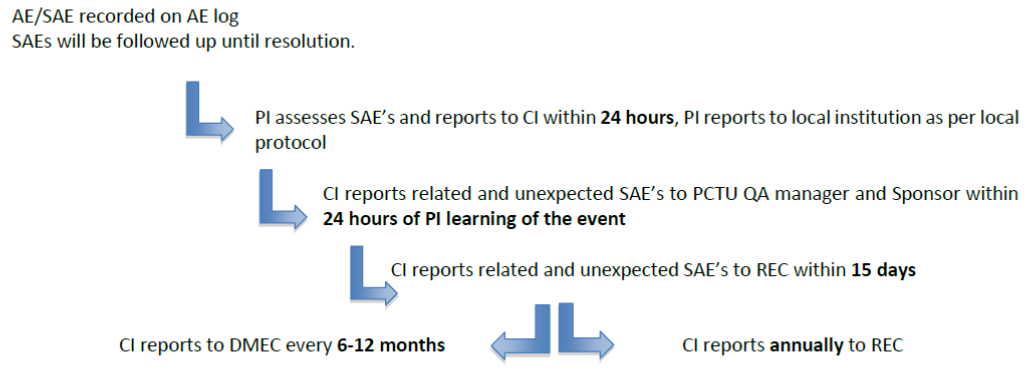

Supplement: Supplementary file 6 — Communication organogram for reporting serious adverse events. (TIF 185 kb) [file 13063_2018_2456_MOESM6_ESM.tif]

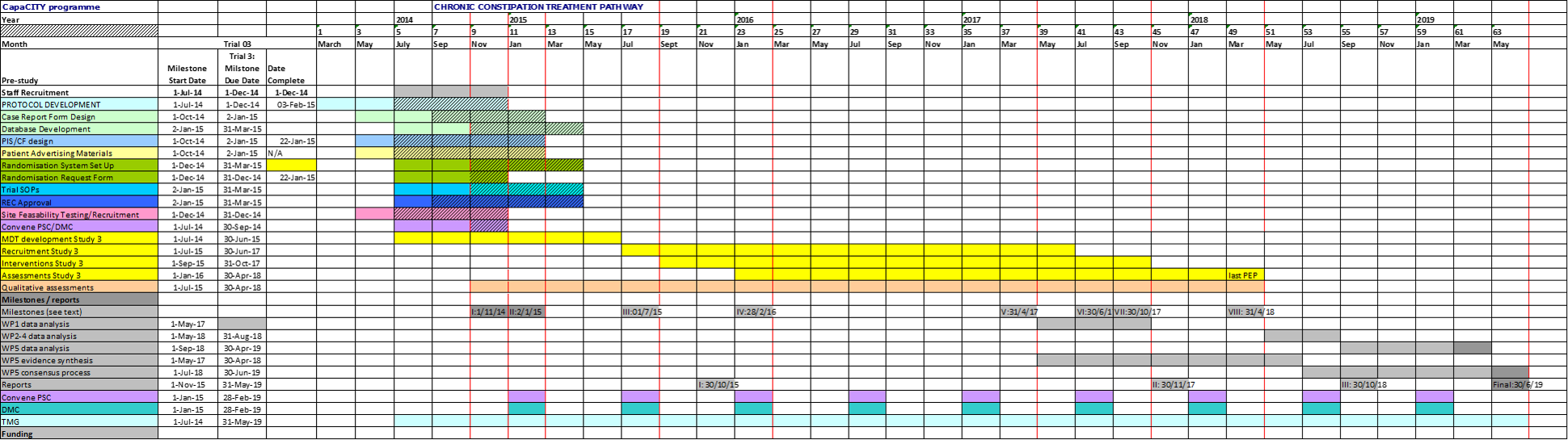

Supplement: Supplementary file 7 — Criteria for quality assessment of laparoscopic ventral mesh rectopexy (LVMR). (TIF 498 kb) [file 13063_2018_2456_MOESM7_ESM.tif]
