# Supplementary material for: Stepped-wedge randomised trial of laparoscopic ventral mesh rectopexy in adults with chronic constipation: study protocol for a randomized controlled trial
Source: Trials. 2018 Feb 5;19:90. doi: 10.1186/s13063-018-2456-3 (PMC5800022; doi:10.1186/s13063-018-2456-3)
Supplement: Supplementary file 8 — CapaCiTY study 3 Gantt chart. (DOC 49 kb) [file 13063_2018_2456_MOESM8_ESM.doc]

**Additional file 1: Criteria for quality assessment of laparoscopic ventral mesh rectopexy (LVMR).**

| # | Key surgical steps | Excellent  (4) | Good  (3) | Poor  (2) | Not performed  (1) |
| --- | --- | --- | --- | --- | --- |
| 1 | Patient position |  |  |  |  |
| 2 | Port-site insertion |  |  |  |  |
| 3 | Left retraction of the rectosigmoid junction and peritoneal incision over the right side of the sacral promontory |  |  |  |  |
| 4 | Incision of Denonvillier’s fascia and opening of the rectovaginal septum |  |  |  |  |
| 5 | Mesh insertion and fixation |  |  |  |  |
| 6 | Suture of the pericervical fascia/vaginal vault to the anterior aspect of the mesh |  |  |  |  |
| 7 | Closure of the lateral borders of the incised peritoneum over the mesh. |  |  |  |  |
| 8 | Ports closure |  |  |  |  |
|  | **FINAL SCORE** |  |  |  |  |
|  |  | **Adherence** | | **Non-adherence** | **Failure** |

‘**Adherence**’ to the standardized surgical technique for LVMR will be determined by scoring ‘good’ or ‘excellent’ to all steps.

‘**Non-adherence**’ to the standardized surgical technique for LVMR will be determined by scoring ‘poor’ in only 1 step excepting #6. Notification of error and re-submission required.

‘**Failure**’ to comply with the standardized surgical technique for LVMR will be determined by scoring ‘poor’ in ≥2 steps or not-performing any steps excepting #6. In this case, a mandatory training is required with possible withdrawal of site from the study.
